# The small non-coding RNA response to virus infection in the *Leishmania* vector *Lutzomyia longipalpis*

**DOI:** 10.1371/journal.pntd.0006569

**Published:** 2018-06-04

**Authors:** Flávia Viana Ferreira, Eric Roberto Guimarães Rocha Aguiar, Roenick Proveti Olmo, Karla Pollyanna Vieira de Oliveira, Emanuele Guimarães Silva, Maurício Roberto Viana Sant'Anna, Nelder de Figueiredo Gontijo, Erna Geessien Kroon, Jean Luc Imler, João Trindade Marques

**Affiliations:** 1 Department of Biochemistry and Immunology, Instituto de Ciências Biológicas, Universidade Federal de Minas Gerais, Belo Horizonte, Minas Gerais, Brazil; 2 Department of Microbiology, Instituto de Ciências Biológicas, Universidade Federal de Minas Gerais, Belo Horizonte, Minas Gerais, Brazil; 3 Department of Parasitology, Instituto de Ciências Biológicas, Universidade Federal de Minas Gerais, Belo Horizonte, Minas Gerais, Brazil; 4 Université de Strasbourg, CNRS M3I/UPR9022, Inserm MIR/U1257, Strasbourg, France; Kenya Agricultural and Livestock Research Organization, KENYA

## Abstract

Sandflies are well known vectors for *Leishmania* but also transmit a number of arthropod-borne viruses (arboviruses). Few studies have addressed the interaction between sandflies and arboviruses. RNA interference (RNAi) mechanisms utilize small non-coding RNAs to regulate different aspects of host-pathogen interactions. The small interfering RNA (siRNA) pathway is a broad antiviral mechanism in insects. In addition, at least in mosquitoes, another RNAi mechanism mediated by PIWI interacting RNAs (piRNAs) is activated by viral infection. Finally, endogenous microRNAs (miRNA) may also regulate host immune responses. Here, we analyzed the small non-coding RNA response to *Vesicular stomatitis virus* (VSV) infection in the sandfly *Lutzoymia longipalpis*. We detected abundant production of virus-derived siRNAs after VSV infection in adult sandflies. However, there was no production of virus-derived piRNAs and only mild changes in the expression of vector miRNAs in response to infection. We also observed abundant production of virus-derived siRNAs against two other viruses in *Lutzomyia* Lulo cells. Together, our results suggest that the siRNA but not the piRNA pathway mediates an antiviral response in sandflies. In agreement with this hypothesis, pre-treatment of cells with dsRNA against VSV was able to inhibit viral replication while knock-down of the central siRNA component, Argonaute-2, led to increased virus levels. Our work begins to elucidate the role of RNAi mechanisms in the interaction between *L*. *longipalpis* and viruses and should also open the way for studies with other sandfly-borne pathogens.

## Author summary

Sandflies are important insect vectors that transmit many species of *Leishmania*, bacteria and viruses. We know very little about how this insect vector responds to viral infection. RNA interference (RNAi) utilizes small non-coding RNAs to regulate different aspects of animal physiology, including immune responses. Small interfering RNAs (siRNAs) mediate a major antiviral response in insects. Virus-derived PIWI-interacting RNAs (piRNAs) can also be generated during infection, at least in some insects. Finally, endogenous microRNAs (miRNA) can regulate the host response to infection. Here we show that virus infection triggers activation of the siRNA pathway but not production of piRNAs in the sandfly *Lutzomyia longipalpis*. Furthermore, activation or inhibition of the siRNA pathway had a direct effect on viral replication. We also show that virus infection caused mild changes to the expression of endogenous miRNAs. Our work describes for the first time a model to study virus infection in sandflies and highlights the importance of the siRNA pathway for the control of virus infection in *L*. *longipalpis*. The framework described here can be used to explore other aspects of the vector-pathogen interactions.

## Introduction

Phlebotomine sandflies (Diptera: Pshychodidae) are important vectors for a wide range of pathogens [1]. Protozoans of the *Leishmania* genus are the most studied of sandfly-borne pathogens but these insects can also transmit bacteria and viruses. Arthropod-borne viruses (arboviruses) transmitted by sandflies are associated with several human and animal diseases, mostly characterized by flu-like symptoms but also some severe cases of encephalitis [2–4]. Sandfly-borne viruses belong to several genera including *Vesiculovirus* (family *Rhabdoviridae)*, *Phlebovirus* (family Bunyaviridae) and *Orbivirus* (family *Reoviridae*) [5–7]. *Vesiculovirus* and *Orbivirus* are mainly restricted to the Americas while *Phlebovirus* are of important concern in Southern Europe and Turkey [7–10]. The *Vesiculovirus* genus includes several human and animal pathogens such as *Vesicular stomatitis virus* (VSV) that causes outbreaks in cattle and horses [11]. Despite the importance of sandfly borne viruses, viral infections remain poorly studied in this vector. Current laboratory models to dissect sandfly-pathogen interactions are mostly restricted to *Leishmania* [12–14] while most information on the transmission of viruses is based on field studies [6, 8, 15, 16].

Arboviruses need to overcome physical and immunological barriers to replicate in the insect vector and be transmitted to the vertebrate host. One of the most important antiviral immune responses in insects is mediated by RNA interference (RNAi). RNAi refers to different mechanisms of regulation of gene expression mediated by small non-coding RNAs [17]. The small interfering RNA (siRNA) is considered the main antiviral response in insects [18, 19]. This pathway is activated by double stranded RNA produced during viral replication that is processed by the RNaseIII enzyme Dicer-2 into 21 nucleotide (nt) siRNAs [20]. These siRNAs are then loaded by Dicer-2 and the small dsRNA binding protein r2d2 onto the nuclease Argonaute- 2 (AGO2) to form the RNA-induced silencing complex (RISC) that destroys complementary targets [21]. The PIWI-interacting RNA (piRNA) pathway can also be activated during viral infection although it is restricted to certain viruses and insect hosts [22–24]. In this case, the biogenesis of piRNAs does not require dsRNA specific nucleases such as Dicers. Rather, single stranded piRNA precursors are processed by different nucleases such as Zucchini nuclease and PIWI proteins to generate 24–29 nt small RNAs [25]. In addition to RNAi mechanisms that can directly target the virus, host small RNAs may also participate in the immune responses by regulating the expression of endogenous genes. MicroRNAs (miRNA) are endogenous 22 nt small RNAs that control the expression of hundreds of genes [26, 27]. A large number of immune genes are regulated by host miRNA that can be affected by infection. The majority of our knowledge about the small RNA response to infection in insects comes from studies in *D*. *melanogaster* and mosquitoes [22, 24, 28–36]. Currently, there is only one study about small RNAs in sandflies by our group, which suggested that the siRNA pathway was not activated by viruses in *L*. *longipalpis* [23].

Here, we develop a laboratory model to dissect virus infection in *L*. *longipalpis* utilizing VSV that is transmitted by sandflies in the wild [2]. Using this model, we analyzed the global small non-coding RNA response to virus infection. We found that VSV strongly induced the production of siRNAs but not piRNAs in adult sandflies. We further demonstrate that this pattern is conserved in response to two novel viruses found in Lulo, a cell line derived from *L*. *longipalpis*. Interestingly, VSV did not induce significant changes the profile of vector miRNAs, which is in agreement with the mild effects observed in infected cells and adult insects. Our data are the first to characterize the small RNA response to viruses and the antiviral role of RNAi in sandflies using a relevant model of VSV infection. This study should open the way for more in depth studies of the role of RNAi in regulating immune responses in sandflies as well as the tripartite interaction between vector, viruses and other pathogens such as *Leishmania*.

## Results

### Characterization of VSV infection in *L*. *longipalpis*

**Fig 1:**
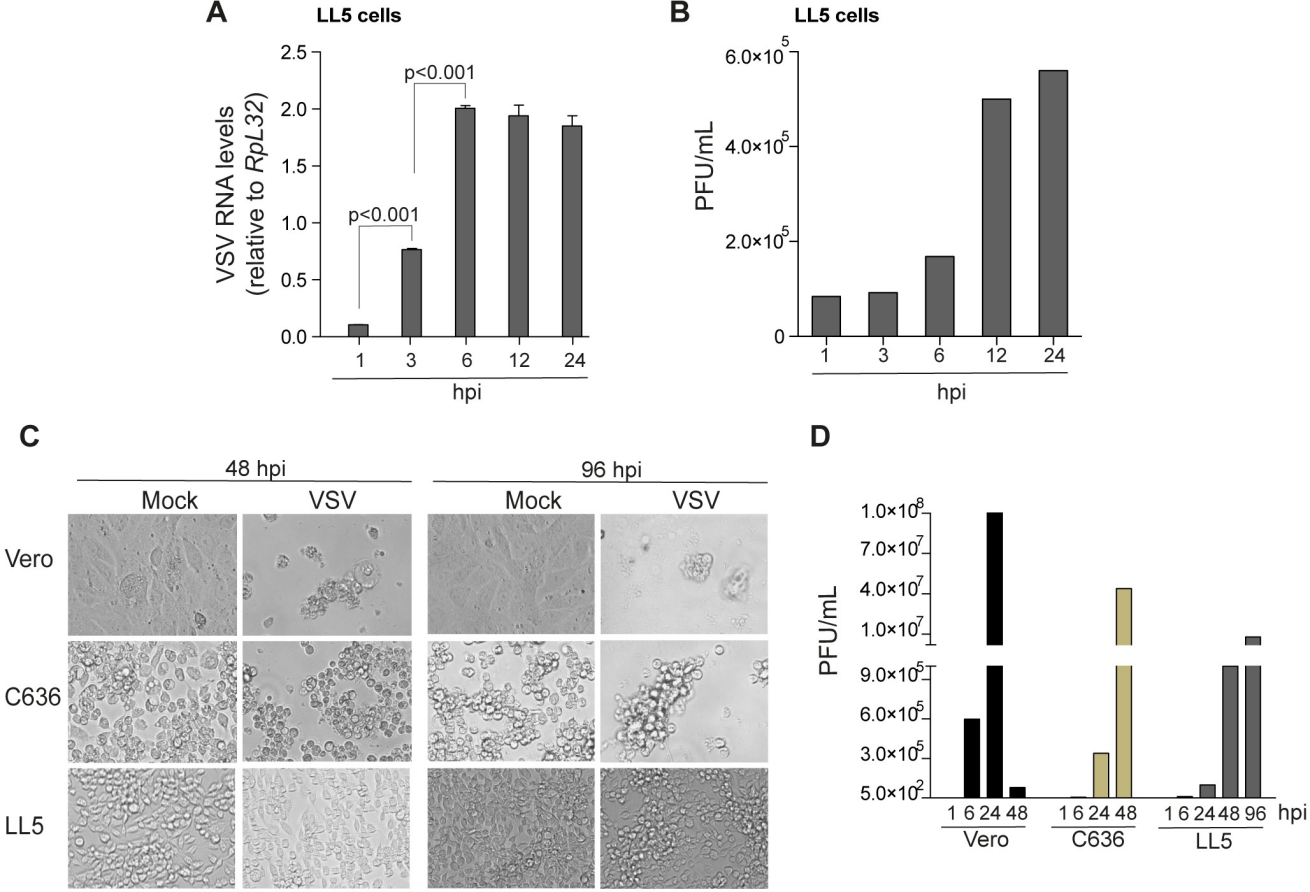
VSV replication in *Lutzomyia* cell lines. **(A)** Kinetics of VSV replication in LL5 cell using a MOI of 10 PFU/mL. Statistical significance was determined using Tukey’s Multiple Comparison test between infected groups at different times post infection. This experiment is representative of three biological replicates. Significant p values are indicated in the figure. **(B)** Infectious VSV particles produced in culture supernatants of LL5 cells were assayed by PFU in Vero cell. This experiment is representative of three biological replicates **(C)** Analysis of cell monolayers at different times after VSV infection in Vero, C636 and LL5 cells. **(D)** Infectious viral particles produced in the supernatant at different points after VSV infection in Vero, C636 and LL5 cells with a MOI of 0.1 PFU/mL. Experiments are representative of at least two biological replicates.

Previous studies have suggested that sandflies play a role in maintenance and transmission of VSV in nature [7, 16, 37–40]. In order to explore the interaction between VSV and sandflies, we initially characterized viral replication in cultured LL5 cells derived from *L*. *longipalpis*. Dose-response experiments demonstrated that using different multiplicities of infection (MOI) 0.4, 2 and 10 plaque-forming units (PFU)/cell, VSV achieved the same levels of viral RNA at 24 hours post infection (hpi) (**S1 Fig**). Using 10 PFU/cell, we observed that VSV RNA levels increased early after infection and reached a plateau at 6 hpi that remained similar until 24 hpi (**Fig 1A**). We also observed that infectious viral particles and viral RNA were detected in supernatant of LL5 cells during early hours of infection (**Fig 1B and S1 Fig**). Despite clear viral replication, we observed no obvious cytopathic effects (CPE) in LL5 cells after VSV infection (**Fig 1C**). In contrast, mammalian cell lines (Vero) were completely destroyed by VSV infection (**Fig 1C**). Mosquito C6/36 cells also displayed CPE such as cell rounding after VSV infection (**Fig 1C**). Although the kinetics of viral replication was different, LL5 cells generated a similar number of viral particles compared to Vero and C636 cells (**Fig 1D**). These experiments suggested that VSV productively replicates in *L*. *longipalpis* cells without causing much damage. Similar observations have been reported elsewhere [41].

**Fig 2:**
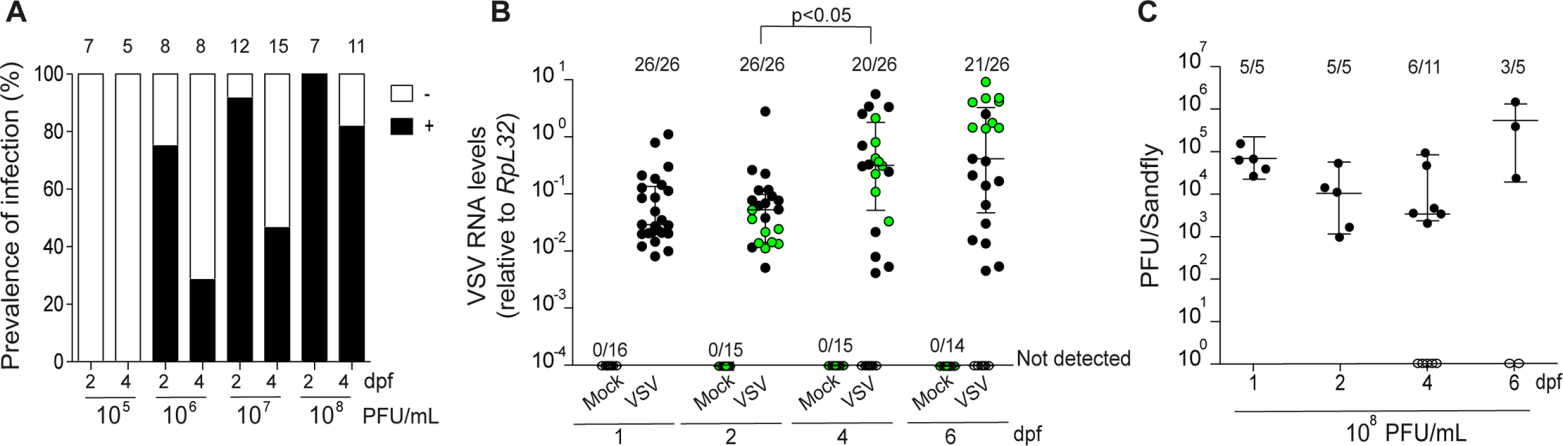
VSV replication in adult *L*. *longipalpis*. **(A)** Adult *L*. *longipalpis* sandflies were fed with blood containing different concentrations of VSV and the prevalence of infection was analyzed by RT-qPCR at 2 and 4 dpf. The number of individuals tested is indicated at each time point. **(B)** Adult sandflies were given a blood meal containing 10^8^ PFU/mL of VSV and viral RNA levels in individual sandflies were determined at 1, 2, 4 and 6 dpf. The number of infected individuals is indicated at each time point. Control sandflies (Mock) were fed with blood without virus. Black circles indicate individuals with detectable viral RNA levels. Individuals used to prepare small RNA libraries shown in Fig 3 are indicated in green. Scatter dot plot shows median with interquatile ranges. Statistical significance was determined using Dunn’s Multiple Comparison test for infected groups at different times after feeding. Significant *p* values are indicated in the figure. **(C)** *L*. *longipalpis* were given a blood meal with containing 10^8^ PFU/mL of VSV and infectious particles in each individual were assayed by PFU. The number of infected individuals and the total are indicated at each time point. Black circles indicate individuals with detectable infectious particles. Experiments are representative of at least 3 biological replicates.

We next performed a dose-response experiment to analyze the threshold of VSV infection *in vivo*. Adult female *L*. *longipalpis* were fed with blood containing different concentrations of VSV and the infection was monitored at 2 and 4 days post feeding (dpf). These two time points allowed us to analyze the amount of virus in the inoculum before full blood digestion at 2 dpf from productive infection at 4 dpf. In these experiments, we observed that the minimum concentration required to productively infect sandflies was 10^6^ PFU/mL (**Fig 2A**). At this concentration we observed that about 25% of individuals were infected at 4 dpf, after blood digestion. At higher viral concentrations, 10^7^ and 10^8^ PFU/mL in the blood meal, over 40 and 60% of adult sandflies were infected at 4 dpf, respectively (**Fig 2A**). At the highest concentration of VSV in the blood meal, 10^8^ PFU/mL, levels of the viral RNA increased continually from 1 to 6 dpf. Infected sandflies at 6 dpf had 100 fold more VSV RNA than the inoculum detected at 1 dpf (**Fig 2B)**. Infectious viral particles were also detected in sandflies throughout the kinetics of infection *in vivo* (**Fig 2C**). VSV RNA was not detected in mock infected sandflies, which were used as controls (**Fig 2B**). We observed no differences in the mortality of sandflies infected by VSV compared to the control group. These results indicate that our laboratory model reproduces the susceptibility of sandflies to VSV observed in field collected insects [7, 37]. The benign effect of VSV in cells and adult *L*. *longipalpis* indicates that the virus is maintained at tolerable levels by the host. These observations are consistent with sandflies working as vectors for this virus in the wild.

### Activation of the siRNA pathway by virus infection in sandflies

The siRNA pathway is a major antiviral defense mechanism in insects and could be involved in controlling VSV replication in *L*. *longipalpis*. In order to analyze the antiviral response mediated by the siRNA pathway in *L*. *longipalpis*, small RNA libraries were constructed using total RNA from VSV infected individuals at 2, 4 and 6 dpf (indicated in **Fig 2B**). Mock infected individuals were used as controls. Raw sequencing results from libraries prepared from infected and control individuals were similar (**S1 Table**).

**Fig 3:**
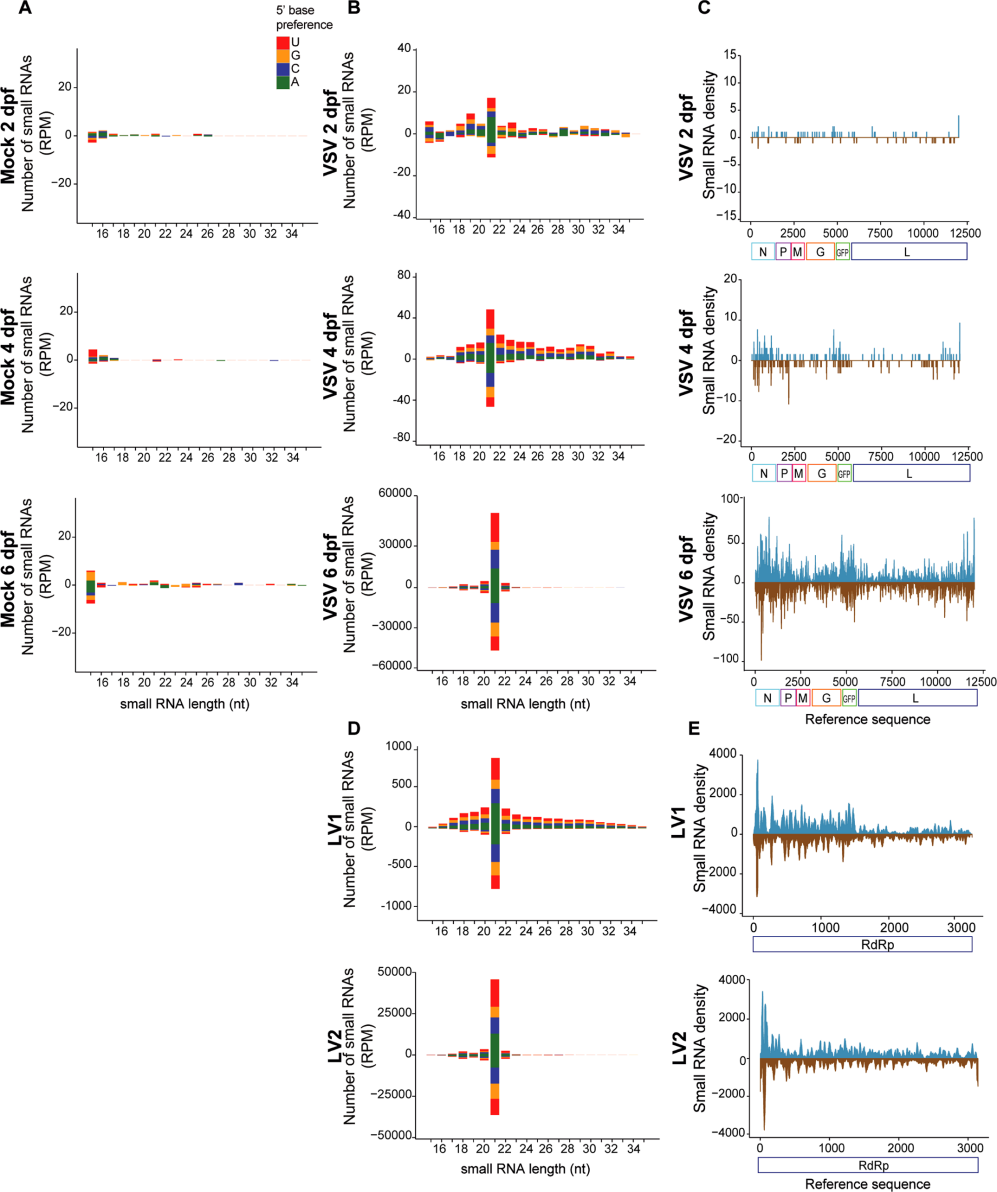
Production of virus-derived small RNAs in *L*. *longipalpis*. **(A)** Analysis of small RNA libraries from control *L*. *longipalpis* (mock) at 2, 4 and 6 dpf shows absence of VSV-derived small RNAs. **(B)** Size distribution of VSV-derived small RNAs at 2, 4 and 6 dpf in infected *L*. *longipalpis* shows a symmetrical peak at 21 nt that is characteristic of siRNAs. **(C)** Coverage of virus-derived small RNAs along the VSV genome from libraries prepared at 2, 4 and 6 dpf shows homogenous and symmetrical distribution. **(D)** Size distribution of small RNAs derived from LV1 and LV2 found in Lulo cells derived from *Lutzomyia* shows a symmetrical peak at 21 nt that is characteristic of siRNAs. **(E)** Coverage of virus-derived small RNAs along the genome of LV1 and LV2 from libraries prepared from Lulo cells shows homogenous and symmetrical distribution. 5’ base preferences of small RNAs are indicated by color. Distribution of small RNAs over sense (blue) and anti-sense (brown) strands of the viral genomes are indicated.

We next analyzed the presence of VSV-derived small RNAs in libraries from infected and control sandflies. Control individuals did not show significant accumulation of virus derived small RNAs (**Fig 3A**). Infected individuals accumulated increasing amounts of VSV-derived small RNAs from 2 to 6 dpf proportionally to the viral load observed at each time point (**Fig 3B**). In infected individuals, the profile of VSV-derived small RNAs showed a clear peak at 21 nt in size, a ratio of ~1 between sense/antisense sequences without a clear 5’ base preference (**Fig 3B**). In addition, the small RNAs mapped across the entire length of the viral genome on both positive and negative strands of the genome (**Fig 3C**). The profile of VSV-derived small RNAs in *L*. *longipalpis* was very similar to the antiviral siRNA response observed in *Drosophila melanogaster* [32, 36]. These results suggest that VSV triggers an antiviral response mediated by the siRNA pathway in sandflies.

In order to investigate whether this was unique to VSV, we sequenced small RNAs from cell lines derived from sandflies and used a metagenomic strategy to identify viruses [23]. In one *L*. *longipalpis* cell line, Lulo, our strategy identified two novel viruses causing persistent infections. Phylogenetic analysis suggested that they belong to two separate genera of RNA viruses, *Luteovirus* and *Alphapermutotetravirus*, and were named *Lulo virus 1 and 2*, respectively (LV1, LV2) (**S2 Fig**). In Lulo cells, both viruses generated small RNAs with a peak of 21 nt in size symmetrically derived from both positive and negative strands of the genome and no 5’ base preferences (**Fig 3D and 3E**). The small RNA profiles observed for LV1 and LV2 are consistent with the activation of siRNA pathway. These results suggest that the siRNA pathway is broadly activated by viral infection in *L*. *longipalpis*. It is important to point out that the Lulo and LL5 cell lines were obtained from embryonic tissues [42, 43] and therefore may not mimic a *bona fide* RNAi response produced by a differentiated cell.

### The expression of siRNA genes is not modulated by VSV infection

**Fig 4:**
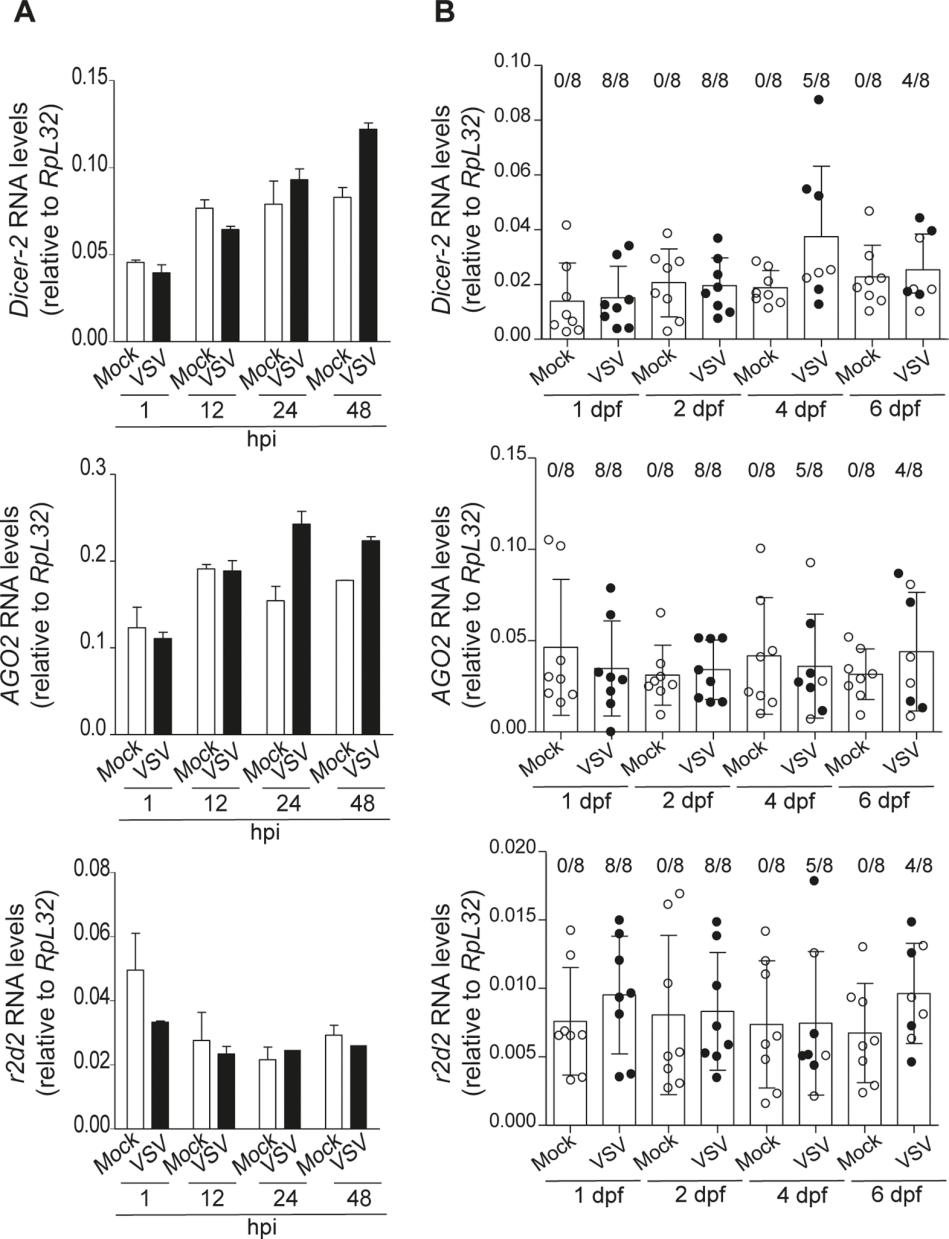
RNAi pathway genes are not modulated by VSV infection in *L*. *longipalpis*. **(A)** Expression of *L*. *longipalpis* genes encoding *Dicer-2*, *AGO2* and *r2d2* in control and VSV-infected LL5 cells at different time points. **(B)** Expression of *L*. *longipalpis* genes encoding *Dicer-2, AGO2 and r2d2* in adult sandflies fed with a blood meal containing VSV. Control sandflies (Mock) were fed with blood without virus. Black circles indicate individuals with detectable viral RNA levels. Numbers of infected individuals and the total are indicated at each time point. No significant differences were observed.

Immune genes are often inducible upon infection in order to optimize the defense response [44, 45]. In a few cases, increased expression of RNAi genes has been observed in insects and other organisms upon viral infection or exposure to dsRNA [46–48]. In order to analyze the expression of siRNA genes in sandflies, we first identified in the genome of *L*. *longipalpis* genes encoding orthologs to core components of the siRNA pathway, *Dicer-2*, *AGO2* and *r2d2*. We next measured RNA levels of these genes in LL5 cells and adult female *L*. *longipalpis* after VSV infection. There were no significant changes in the expression of *Dicer-2*, *AGO2* and *r2d2* in cells between 1 and 48 hpi and sandflies with VSV at 1, 2, 4 and 6 dpf (**Fig 4A and 4B**). These results indicate that similar to most insects, the activity of the siRNA pathway is not transcriptionally regulated during infections in sandflies.

### The siRNA pathway controls VSV infection in *L*. *longipalpis*

**Fig 5:**
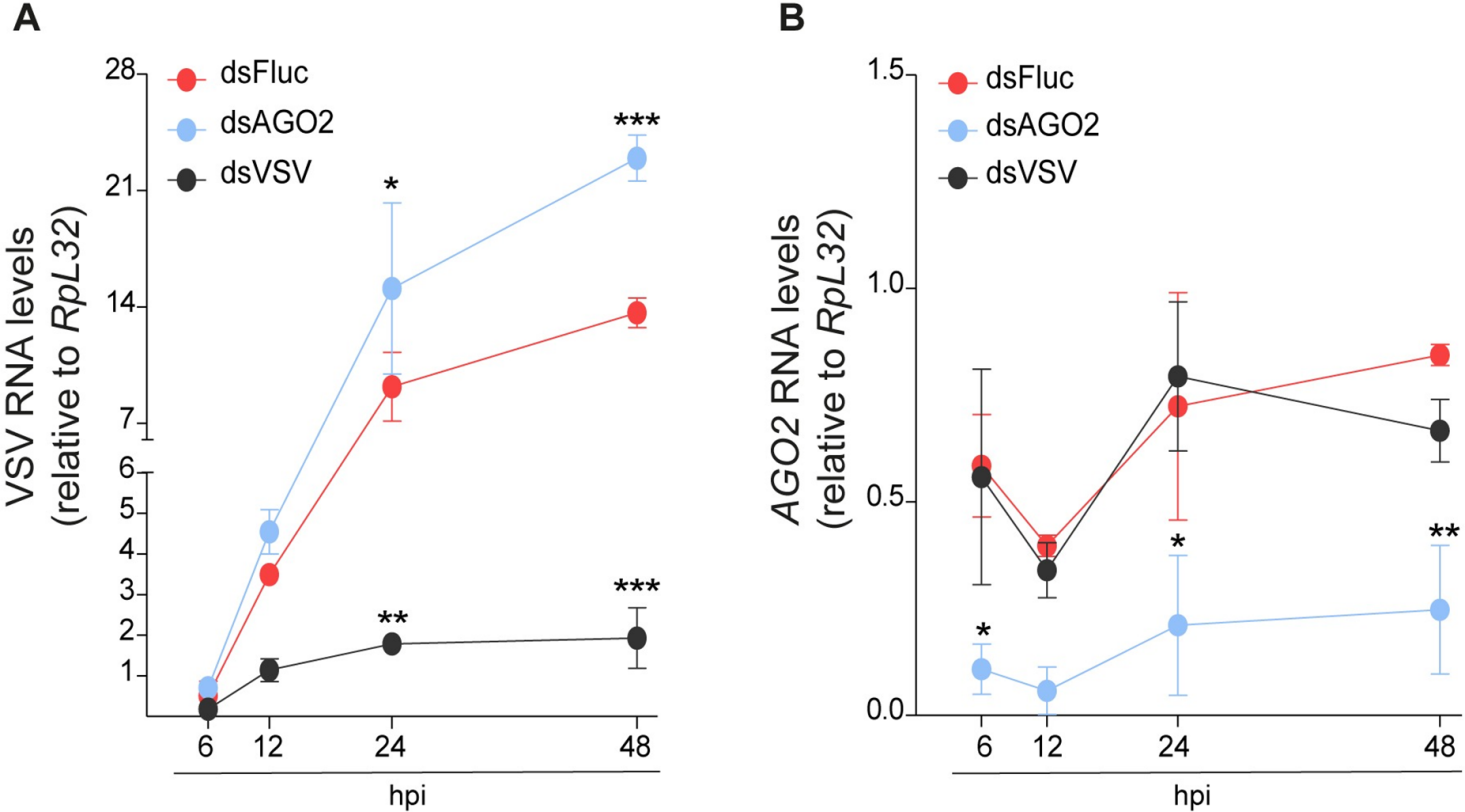
The siRNA pathway controls VSV replication in *L*. *longipalpis* cells. **(A)** LL5 cells were transfected with dsRNA targeting *Firefly* Luciferase (dsFluc), *L*. *longipalpis* *AGO2* (dsAGO2) or VSV (dsVSV). Three days after transfection, cells were infected with 1 PFU/cell of VSV and viral RNA levels were measured by qPCR at different times post infection. **(B)** *AGO2* RNA levels were measured by RT-qPCR in cells treated with dsRNA targeting *Firefly* Luciferase (dsFluc), *L*. *longipalpis AGO2* (dsAGO2) or VSV (dsVSV). Statistical significance was determined using two-way ANOVA test to compare between dsVSV and dsAGO2 groups and the control (dsFluc). Significant *p* values are indicated in the figure (* p<0.05; ** p<0.01; *** p<0.001). Experiments are representative of two biological replicates.

The generation of VSV-derived siRNAs in *L*. *longipalpis* suggested that the siRNA pathway mediates an antiviral response against this virus as observed in *D*. *melanogaster* [32, 36]. In order to investigate whether activation of the siRNA pathway has an antiviral effect in *L*. *longipalpis*, we first transfected LL5 cells with dsRNA complementary to VSV prior to virus infection. Pre-engagement of the siRNA pathway with virus-specific dsRNA led to a significant reduction in viral replication at 24 and 48 hpi compared to control cells treated with a non-related dsRNA targeting the *Firefly* Luciferase gene (**Fig 5A**). We next utilized dsRNA treatment to silence AGO2, the central component of the siRNA pathway, in LL5 cells. dsRNA treatment lead to a significant reduction in *AGO2* levels (**Fig 5B**). As a result, AGO2 silenced cells had significantly increased viral RNA levels compared to controls cells at 24 and 48 hpi (**Fig 5A**). These results indicate that the siRNA pathway has an important role in controlling viral infection in *L*. *longipalpis*.

### Absence of virus-derived piRNAs in *L*. *longipalpis*

**Fig 6:**
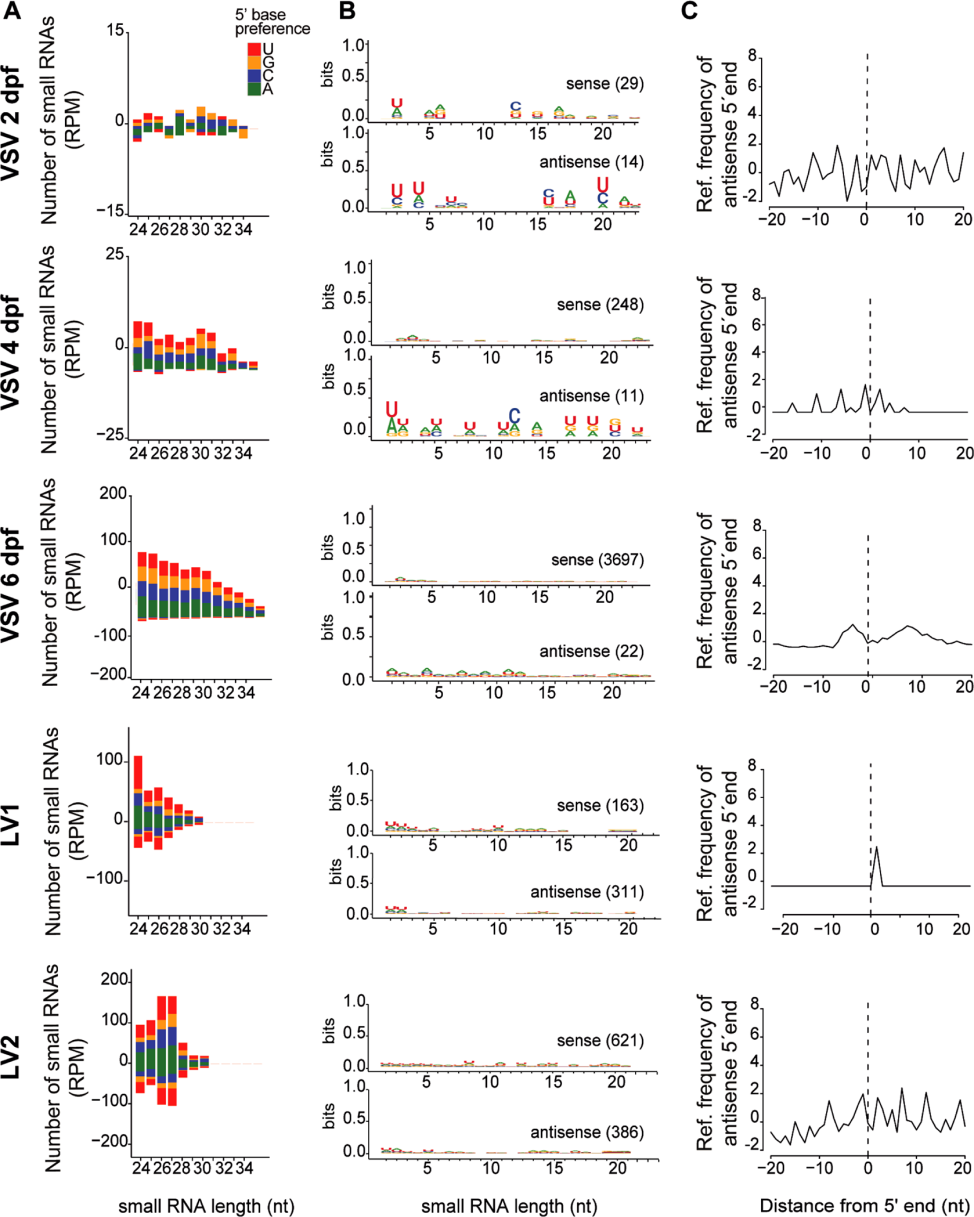
Absence of virus-derived piRNAs in *L*. *longipapis*. **(A)** Size distribution of small RNAs derived from VSV, LV1 and LV2 in the 24 to 35 nt range considering each strand separately. 5’ base preferences are indicated by color. **(B)** Nucleotide preferences for each position of small RNAs between 24–30 nt are shown as a weblogo. The number of reads analyzed is indicated in brackets. **(C)** Relative frequency of overlap between 5’ end of small RNAs between 24–30 nt in opposite strands.

In addition to the activation of the siRNA pathway, production of virus-derived piRNAs has been observed during viral infection in dipterans. *In vivo*, *Aedes* mosquitoes infected with *Chikungunya virus* (CHIKV) and *Phasi Charoen like-virus* induced clear production of piRNAs [22, 23]. Virus-derived piRNAs have been detected in cell lines derived from mosquitoes and also other dipterans such as culicoides and *D*. *melanogaster* [22, 49–51]. However, it remains unclear whether other dipterans generate virus-derived piRNAs *in vivo* since *in vitro* results lack the context of whole animals. For example, despite numerous efforts, virus-derived piRNAs have not been detected in adult *Drosophila* [24]. In this regard, sandflies are closely related to mosquitoes and fruit flies and could help understand whether virus-derived piRNAs appeared only in the mosquito lineage or were lost in *Drosophila*. Thereby we separately analyzed virus-small RNAs in the size range of piRNAs, 24–30 nt, detected both in adult *L*. *longipalpis* and cell lines. We observed accumulation of longer virus-derived small RNAs in adult sandflies infected with VSV and Lulo cells infected with LV1 and LV2 (**Fig 6A**). These small RNAs did not exhibit 1U or 10A enrichment or 10 nt overlap between sense and antisense strands that are considered canonical piRNA characteristics (**Fig 6B and 6C**).

In order to further investigate the absence of virus-derived piRNAs, we re-analyzed previously published small RNA datasets from *L*. *longipapis* infected with three other viruses, LPRV1, LPRV2 and LPNV [23]. These viruses had a broad size distribution of smalls RNAs but none showed characteristics of canonical piRNAs (**S3 Fig**). Together, our results suggest that the piRNA pathway is not engaged by infection with VSV, LV1 and LV2 in *L*. *longipapis*.

### The effect of VSV infection on host miRNAs

In addition to the production of virus-derived small RNAs, modulation of host small RNAs, especially miRNAs, may also occur during viral infection in insects [52]. Thus, we first analyzed the profile of endogenous small RNAs derived from *L*. *longipalpis* in infected and control individuals. We observed no differences in profile of small RNAs between infected and control sandflies. In both cases, the size distribution of small RNAs showed two clear peaks between 21–23 and 24–30 nt representing the typical length of siRNAs/miRNAs and piRNAs, respectively (**S4 Fig**).

**Fig 7:**
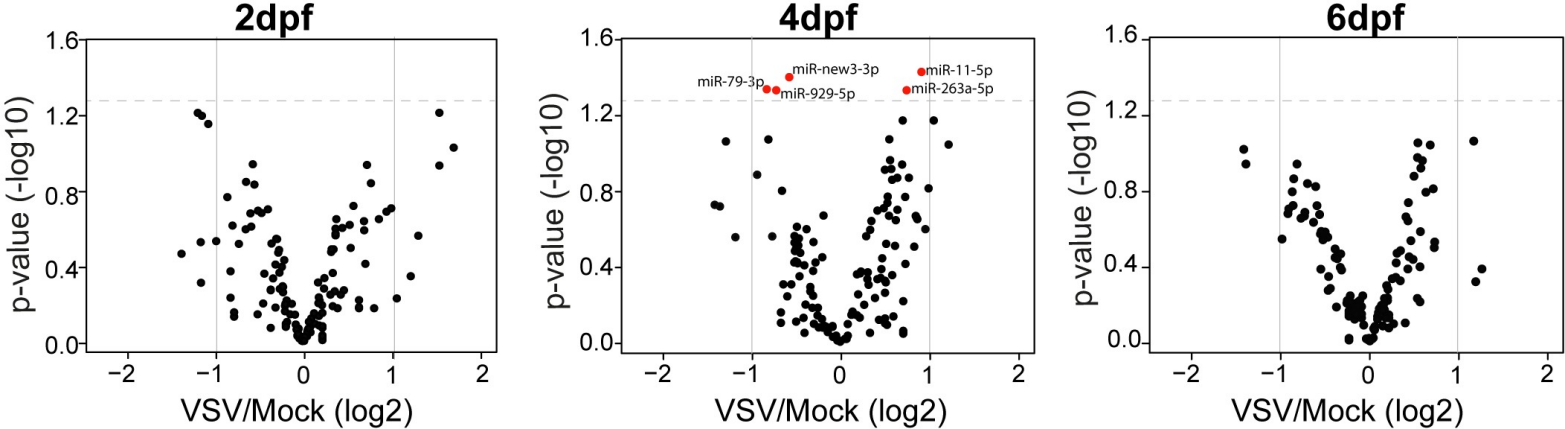
Expression of endogenous miRNAs is mostly unaffected by VSV infection in *L*. *longipalpis*. Volcano plots comparing the expression of miRNAs in adult *L*. *longipalpis* infected with VSV and control individuals at 2, 4 and 6 dpf. At 4dpf, the expression of 5 miRNAs (indicated in red) was significantly (p<0.05) different between infected and control sandflies. At 2 and 6 dpf, there were no significant differences in miRNA expression.

In order to analyze changes in specific small RNAs during VSV infection, we first identified and annotated miRNA genes in the *L*. *longipalpis* genome (data from the sandfly genome consortium). In this effort, we were able to identify 206 miRNAs, most of them conserved in other dipterans such as *D*. *melanogaster* and *A*. *aegypti*. Using this reference, we analyzed differential expression of miRNAs in sandflies infected with VSV compared to control individuals at 2, 4 and 6 dpf. At 2 and 6 dpf, we observed no significant differences in the expression of *Lutzomyia* miRNAs between infected and control individuals. At 4 dpf, we detected 5 miRNAs that were significantly modulated by VSV infection (**Fig 7**). llo-miR-11-5p and llo-miR-263a-5p were up-regulated in VSV infected individuals while llo-miR-new3-3p, llo-miR-929-5p and llo-miR-79-3p were down-regulated. However, it is noteworthy that the magnitude of changes in miRNA expression was smaller than 2-fold between infected and control individuals. Together, these results show that VSV infection causes mild changes in host miRNAs and other endogenous small RNAs. However, it is possible that more pronounced changes might be observed if specific tissues are analyzed.

## Discussion

Here, we successfully developed a laboratory model to dissect the interaction between sandflies and viruses. Sandflies are well recognized as vectors for species of *Leishmania* but we lack information about host-pathogen interactions in the context of viruses. This work is, to the best of our knowledge, the first to analyze antiviral responses against arboviruses in sandflies. Here, we focused in the characterization of RNA interference mechanisms since they mediate powerful antiviral responses in insects such as fruit flies and mosquitoes [22, 24, 28–30, 32, 34–36]. In this regard, our work in sandflies is also an important contribution since most of our understanding about insect small RNA responses to virus infection is based on fruit flies and mosquitoes.

Our results indicate that the siRNA pathway has a conserved antiviral role in sandflies despite previous result from our own group that suggested the absence of virus-derived siRNAs in *Lutzomyia* [23]. Here, we observed that virus-derived siRNAs are produced *in vivo* and *in vitro* and pre-engagement of the siRNA pathway had a direct effect on VSV replication. Furthermore, silencing of AGO2 in *Lutzomyia* cells leads to increased viral replication, which suggests they fail to control infection. This antiviral role of the siRNA pathway in sandflies is in agreement with numerous observations in insects and other invertebrates [32, 53, 54]. The broad size profile of small RNAs derived from the viruses we described before suggest that they had the ability to inhibit the siRNA pathway in *L*. *longipalpis* rather than the absence of such response [55]. We note that most studies on the natural antiviral role of RNAi in vector insects have focused on arboviruses that have positive-stranded RNA genomes [22, 34, 35, 56, 57]. VSV is a negative stranded RNA virus that is controlled by the siRNA pathway during artificial infections in *Drosophila* [32, 36]. Our data show that this is also the case in sandflies that are natural vectors for this arbovirus in the wild.

The conspicuous absence of virus-derived piRNAs in sandflies suggests that viruses do not engage the piRNA pathway in these insects. Previous results have indicated that viruses activate the piRNA pathway in mosquitoes but not fruit flies, at least *in vivo* [22, 24]. Sandflies provide an important evolutionary perspective since they are closely related to mosquitoes and fruit flies. Therefore, activation of the piRNA pathway by virus infection seems to have been an acquired characteristic of the mosquito lineage although its role in antiviral defense remains unclear. However, it is still possible that activation of the piRNA pathway is limited to certain viruses or restricted to sandfly tissues that were not analyzed in our model.

In addition to activation of siRNA and piRNA pathways targeting the virus, we investigated whether host miRNAs changed in response to infection. Modulation of specific host miRNAs during infection has been observed in insects, which may be part of a coordinated host response to infection or a consequence of viral replication [58]. However, we observed that VSV induced little changes in the expression of host miRNAs, which is consistent with this infection being well tolerated by sandflies.

Finally, our model can be further used to dissect more complex interactions involving multiple sandfly-borne pathogens and understand other aspects of immune responses. Notably, epidemiologic data showed strong association between transmission of *Leishmania* and *Phlebovirus* in Southern France [59]. This result suggests that viruses may affect sandfly vectorial capacity for *Leishmania* and vice-versa. Viruses may decrease host fitness and affect the ability of sandflies to transmit other pathogens. Good vectors need to tolerate infections to prevent loss of fitness and maintain pathogen levels to allow transmission to a vertebrate host. Our work should allow further studies to dissect the possible effects of tripartite vector-virus-*Leishmania* interactions in sandflies. It is also important to point out that our work is also the first description of small RNAs in sandflies. This opens the doors for future studies on RNAi mechanisms in other contexts such as the role of specific miRNAs during *Leishmania* infection.

## Methods

### Cell lines

Vero cells obtained by ATCC (Maryland, USA) were grown in Dulbecco's Modified Eagle's Medium (DMEM) (Life Technologies) supplemented with 5% FBS and penicillin/ streptomycin (final concentration 100 units/mL, 100 μg/ mL, respectively) at 37°C/ 5% CO2. *Lutzomyia longipalpis* embryonic LL5 [42] and Lulo [43] cells were a kind gift from Dr. André Pitaluga (Fiocruz–Rio Janeiro). Both cells and *Aedes albopictus* C6/36 cells were grown in L-15 medium (SIGMA—Aldrich) supplemented with 10% fetal bovine serum (FBS) (GIBCO) and 1% penicillin/ streptomycin (final concentration 100 units/mL, 100 μg/mL respectively, Life Technologies), at 28°C.

### Viruses

VSV *Indiana virus* expressing green fluorescent protein (GFP) were a kind gift from Dr. Curt Horvath (Northwestern University). Stocks were prepared in Vero cells and virus titrations were done by plaque assay.

### Plaque assay in mammalian cell

Six-well plates containing Vero monolayers with approximately 90% confluence were inoculated with virus and incubated at 37°C for 1h in an atmosphere supplemented with 5% of CO_2_. DMEM supplemented with 2% FBS was added to each well in a volume sufficient to maintain cell monolayers during the subsequent incubation period of 72h at 37°C in atmosphere supplemented with 5% of CO_2_. Vero monolayers were fixed with formalin at 10% (MERCK MILLIPORE, USA) and stained with crystal violet solution at 1% (SYNTH, Brazil) allowing naked eye observation of cytopathic effects. All samples were tested in triplicate.

### Infection of LL5 *Lutzomyia* cells with VSV

LL5 cells (3x10^6^ per well) were seeded in plaque one day prior to infection and infected with VSV at a multiplicity of infection (MOI) of 10 by directly adding the virus to the culture medium. One hour post infection the culture medium was removed, the cells washed with PBS followed by new culture medium addition. The cells were maintained at 25°C and the cells and supernatants were harvested for RNA isolation and plaque assay.

### Transfection of LL5 *Lutzomyia* cells with dsRNA

dsRNA targeting *L*. *longipalpis AGO2*, VSV and *Firefly* luciferase were synthesized using the T7 and SP6 megascript kit. 7 μg of dsRNA was transfected into LL5 cells using Cellfectin (Invitrogen) according to the manufacturer’s protocol. 3 days after transfection, cells were infected with a MOI of 1 PFU/cell of VSV.

### Laboratory rearing of *Lutzomyia longipalpis* sandflies

The sandflies used in this study were obtained from a colony reared in the laboratory that was originally started from individuals from Teresina, Brazil, and maintained at the Laboratory of Physiology of Hematophagous Insects (Department of Parasitology, Instituto de Ciências Biológicas, Universidade Federal de Minas Gerais). Adult sandflies were fed a 70% sucrose solution (w/v) and blood-fed with anesthetized (ketamine, 200 mg/kg) hamsters to trigger egg development. After oviposition, eggs were collected and reared to preserve the colony. All larval instars were fed a crushed mixture of rabbit feces, rabbit chow, and garden soil. Third and fourth instars were supplemented with a mixture of white soy protein and cereal flakes (1:1). The insects were maintained at 27°C, humidity of 80–95%, and a photoperiod schedule of 12 h light/12 h dark.

### Infection of adult sandflies with VSV

Three-day-old female sand flies and private of sugar solution food for a period of 24h were used in this study. The insects were fed with heparinized blood through chick skin membrane attached in an artificial feeder (Hemotek). The artificial infection was performed with blood containing VSV or a control without virus at 37°C. Fully engorged females were selected and harvested individually at 2, 4 and 6 dpf. Insects were anesthetized with carbon dioxide and directly ground in TRIzol (Invitrogen) using glass beads as previously described [23].

### Ethics statement

All procedures involving animals were approved by the ethical review committee of the Universidade Federal de Minas Gerais (CEUA 33/2016 to M.R.V.S).

### Analysis of viral RNA replication and gene expression by RT-qPCR

Total RNA was extracted from individual insects and cells using Trizol reagent according to the manufacturer’s protocol (Invitrogen). 1 μg of total RNA was reverse transcribed using 250 ng of random primers specific primers per reaction. The resulting cDNA was used as template for qPCR reaction containing SYBR Green (Invitrogen) and primers specific for the amplification of the genes of interest. The relative amount of the indicated RNAs normalized to an internal control (*Rpl32*) was calculated using the delta Ct method. Reverse transcription reactions were performed in the absence of primers or enzyme as negative controls for qPCR to ensure the identity of the products. The endogenous gene *Rpl32* qPCR was used as normalization standard. Oligonucleotides designed in this study are described in **S2 Table**.

### Small RNA library construction

For libraries construction, individual insects were first tested for the presence and levels of VSV by RT-qPCR. RNA samples were then pooled according to the viral load in each insect. Total RNA extracted from adult insects or cell lines were used from the construction of small RNA libraries. Small RNAs were selected by size (~18–30 nt) on a denaturing PAGE before being used for construction of libraries as described [36]. All sequencing runs were performed at the IGBMC Microarray and Sequencing platform, a member of the ‘France Génomique’ consortium (ANR-10-INBS-0009), as 1 × 50 base pairs using a HiSeq 2500 instrument. Accession numbers of all libraries described in this study are in **S1 Table**.

### Reference genome

Reference genome of *L*. *longipalpis* (Jacobina strain, version J1.2) was downloaded from the VectorBase website (www.vectorbase.com). The accession number KU721836 was the reference genome of VSV.

### Identification of core genes in the siRNA pathway in the *L*. *longipalpis* genome

Nucleotide sequences of core siRNA genes from *D*. *melanogaster* (*Dicer-2*, *AGO2*, and *r2d2*) were used as query to identify putative homologs in the genomes of *L*. *longipalpis* using SoftBerry software [60]. Putative orthologs were analyzed for domain conservation in predicted protein by using InterProScan [61]. Gene IDs are shown in **S3 Table.**

### Analysis of miRNA expression

miRNA genes in *L*. *longipalpis* were identified using mirDeep [62]. miRNA genes were annotated as part of sandfly genome consortium. Small RNA libraries were mapped against miRNA genes annotated in the *L*. *longipalpis* genome. Bowtie [63] was used for mapping allowing 0 mismatches in the seed and 1 mismatch in the rest of sequence. miRNA counts were normalized and used to evaluate differential expression among samples using R with package DESeq2 considering *p*<0.05 [64].

### Statistical analysis

Pattern-based analysis, small RNA size profile, 5’ base enrichment, density of coverage and additional data analysis were evaluated using in-house Python, Perl and R scripts. Statistics of 5’ base enrichment was calculated as described [32]. All of the experiments were analyzed for statistical significance using the software GraphPad Prism. The Shapiro-Wilk normality test was first applied. Kruskall Wallis test was used for multiple comparisons. *p*<0.05 was considered statistically significant.

## Supporting information

S1 Fig VSV replication in *L*. *longipalpis* LL5 cells. **(A)** Viral RNA levels measured using RT-qPCR in *L*. *longipalpis* LL5 cells using MOIs of 0.4, 2 and 10 PFU/cell at different times after infection. **(B)** Viral RNA detected in the supernatant of the same cells by *qRT-PCR*. Experiments are representative of at least 2 biological replicates.
(PDF)

S2 Fig Phylogeny of viruses identified in *L*. *longipalpis* Lulo cells. Two viruses identified by small RNA sequencing in *L*. *longipalpis* Lulo cells were analyzed by phylogeny. **(A)** *Lulo virus 1* (LV1) clustered with viruses from the *Luteoviridae* family (indicated in orange) and with some unclassified viruses (indicated in purple). **(B)** *Lulo virus 2* (LV2) clustered with viruses from the *Permutotetraviridae* family (indicated in dark blue). Nucleotide sequences were aligned using Muscle implemented in MEGA [65] constructed with Maximum likelihood and applying Poisson model tested using 100 bootstrap replicates. Bootstrap values above 70 are indicated.
(PDF)

S3 Fig Virus-derived small RNAs do not show canonical characteristics of piRNAs in *L*. *longipalpis*. Small RNAs derived from *Lutzomyia Piaui nodavirus* (LPNV), *Lutzomyia Piaui reovirus* 1 (LPRV1) and *Lutzomyia Piaui reovirus 2* (LPRV2) in *L*. *longipalpis* were analyzed for piRNA characteristics. **(A)** The size distribution of small RNAs in the 24–35 nt range considering each strand separately. 5’ base preferences of small RNAs are indicated by color. **(B)** Nucleotide preferences for each position of virus-derived small RNAs between 24–30 nt are shown as a weblogo. **(C)** The relative frequency of overlap between 5’ ends of small RNAs between 24–30 nt in opposite strands is shown.
(PDF)

S4 Fig The general profile of endogenous small RNAs does not change in response to VSV infection in *L*. *longipalpis*. Size distribution of host small RNAs from *L*. *longipalpis* obtained from VSV-infected and control (Mock) sandflies at day 2, 4 and post blood feeding. 5’ base preferences of small RNAs are indicated by color.
(PDF)

S1 Table Overview of small RNA libraries from *L*. *longipalpis*. (DOCX)

S2 Table Oligonucleotides utilized in this study. (DOCX)

S3 Table Accession numbers of siRNA pathway genes from *L*. *longipalpis*. (DOCX)
