# Supplementary material for: The small non-coding RNA response to virus infection in the Leishmania vector Lutzomyia longipalpis
Source: PLoS Negl Trop Dis. 2018 Jun 4;12(6):e0006569. doi: 10.1371/journal.pntd.0006569 (PMC6002125; doi:10.1371/journal.pntd.0006569)
Supplement: S1 Fig — (A) Viral RNA levels measured using RT-qPCR in L. longipalpis LL5 cells using MOIs of 0.4, 2 and 10 PFU/cell at different times after infection. (B) Viral RNA detected in the supernatant of the same cells by qRT-PCR. Experiments are representative of at least 2 biological replicates. (PDF) [file pntd.0006569.s001.pdf]

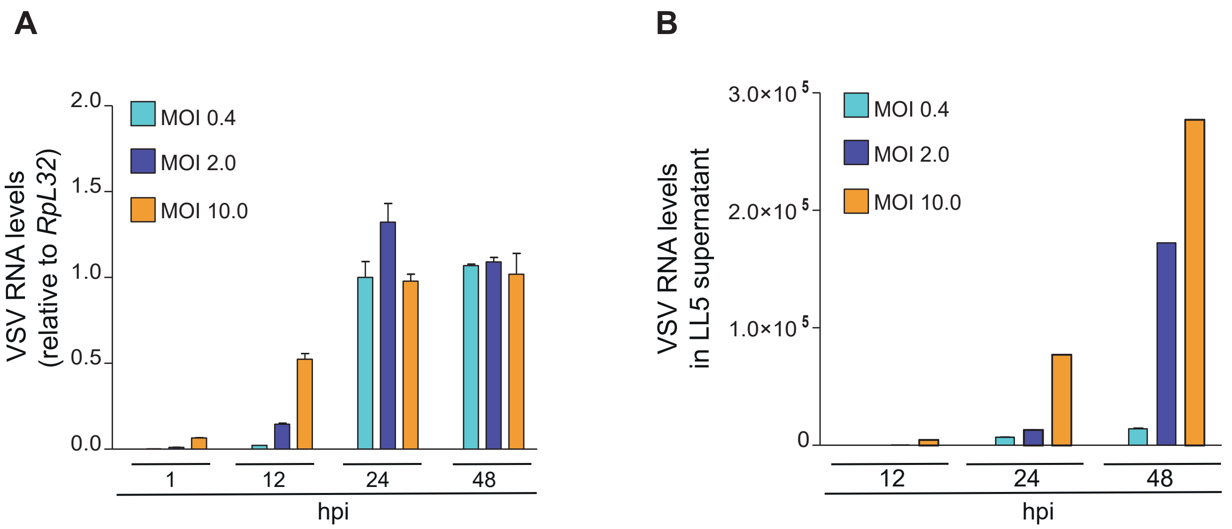

S1 Fig. VSV replication in *L. longipalpis* LL5 cells. (A) Viral RNA levels measured using RT-qPCR in *L. longipalpis* LL5 cells using MOIs of 0.4, 2 and 10 PFU/cell at different times after infection. **(B)** Viral RNA detected in the supernatant of the same cells by RT-qPCR. Experiments are representative of at least 2 biological replicates.
