# Supplementary material for: The small non-coding RNA response to virus infection in the Leishmania vector Lutzomyia longipalpis
Source: PLoS Negl Trop Dis. 2018 Jun 4;12(6):e0006569. doi: 10.1371/journal.pntd.0006569 (PMC6002125; doi:10.1371/journal.pntd.0006569)
Supplement: S2 Fig — Two viruses identified by small RNA sequencing in L. longipalpis Lulo cells were analyzed by phylogeny. (A) Lulo virus 1 (LV1) clustered with viruses from the Luteoviridae family (indicated in orange) and with some unclassified viruses (indicated in purple). (B) Lulo virus 2 (LV2) clustered with viruses from the Permutotetraviridae family (indicated in dark blue). Nucleotide sequences were aligned using Muscle implemented in MEGA [65] constructed with Maximum likelihood and applying Poisson model tested using 100 bootstrap replicates. Bootstrap values above 70 are indicated. (PDF) [file pntd.0006569.s002.pdf]

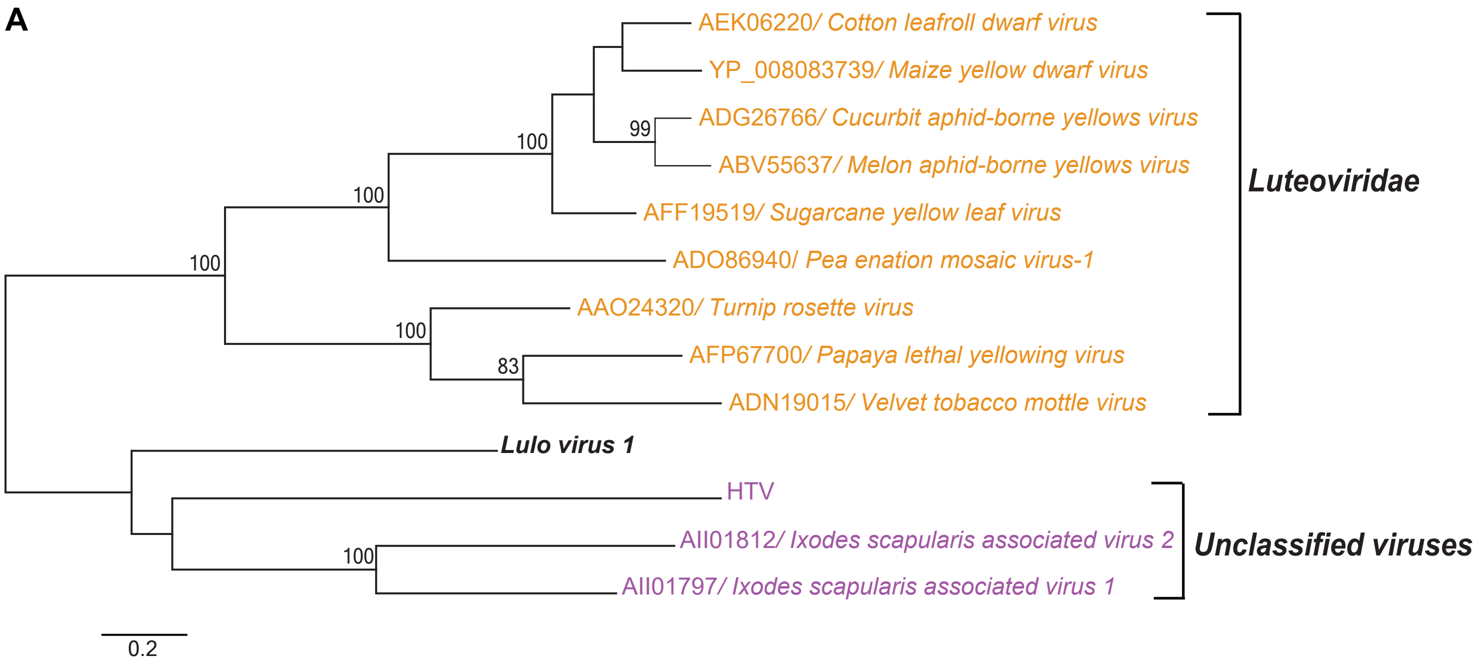

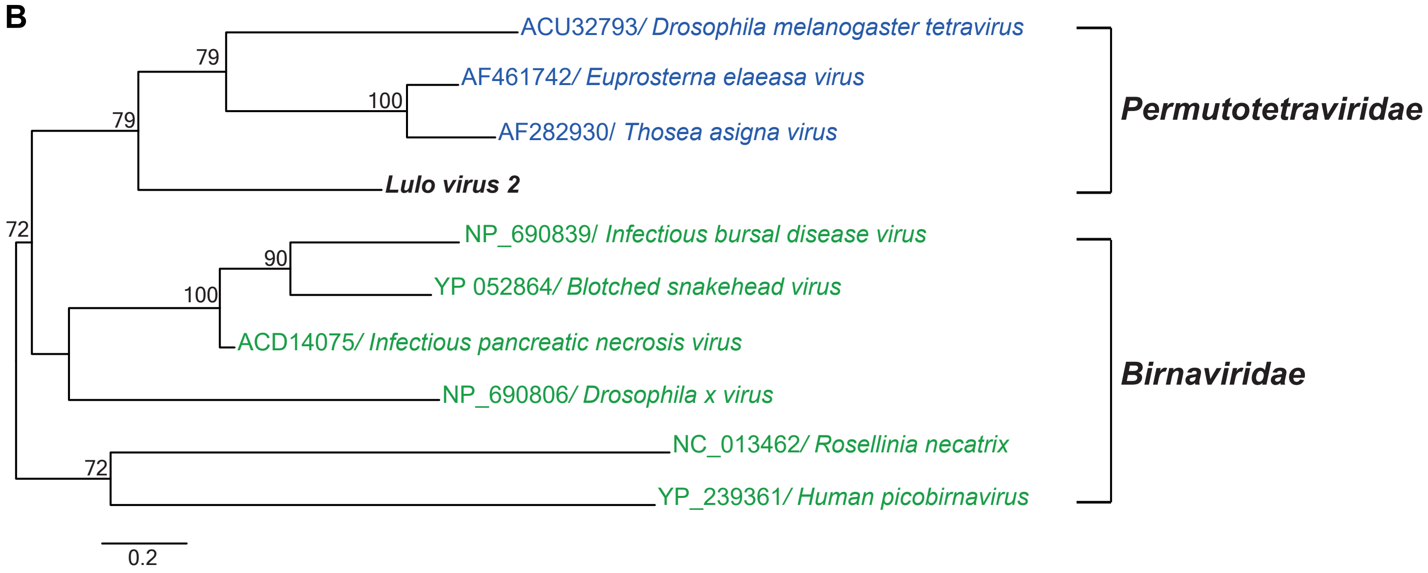

S2 Fig. Phylogeny of viruses identified in *L. longipalpis* Lulo cells. Two viruses identified by small RNA sequencing in *L. longipalpis* Lulo cells were analyzed by phylogeny. **(A)** *Lulo virus 1* (LV1) clustered with viruses from the *Luteoviridae* family (indicated in orange) and with some unclassified viruses (indicated in purple). **(B)** *Lulo virus 2* (LV2) clustered with viruses from the *Permutotetraviridae* family (indicated in dark blue). Nucleotide sequences were aligned using Muscle implemented in MEGA [63] constructed with Maximum likelihood and applying Poisson model tested using 100 bootstrap replicates. Bootstrap values above 70 are indicated.
