# Supplementary material for: The small non-coding RNA response to virus infection in the Leishmania vector Lutzomyia longipalpis
Source: PLoS Negl Trop Dis. 2018 Jun 4;12(6):e0006569. doi: 10.1371/journal.pntd.0006569 (PMC6002125; doi:10.1371/journal.pntd.0006569)
Supplement: S3 Fig — Small RNAs derived from Lutzomyia Piaui nodavirus (LPNV), Lutzomyia Piaui reovirus 1 (LPRV1) and Lutzomyia Piaui reovirus 2 (LPRV2) in L. longipalpis were analyzed for piRNA characteristics. (A) The size distribution of small RNAs in the 24–35 nt range considering each strand separately. 5’ base preferences of small RNAs are indicated by color. (B) Nucleotide preferences for each position of virus-derived small RNAs between 24–30 nt are shown as a weblogo. (C) The relative frequency of overlap between 5’ ends of small RNAs between 24–30 nt in opposite strands is shown. (PDF) [file pntd.0006569.s003.pdf]

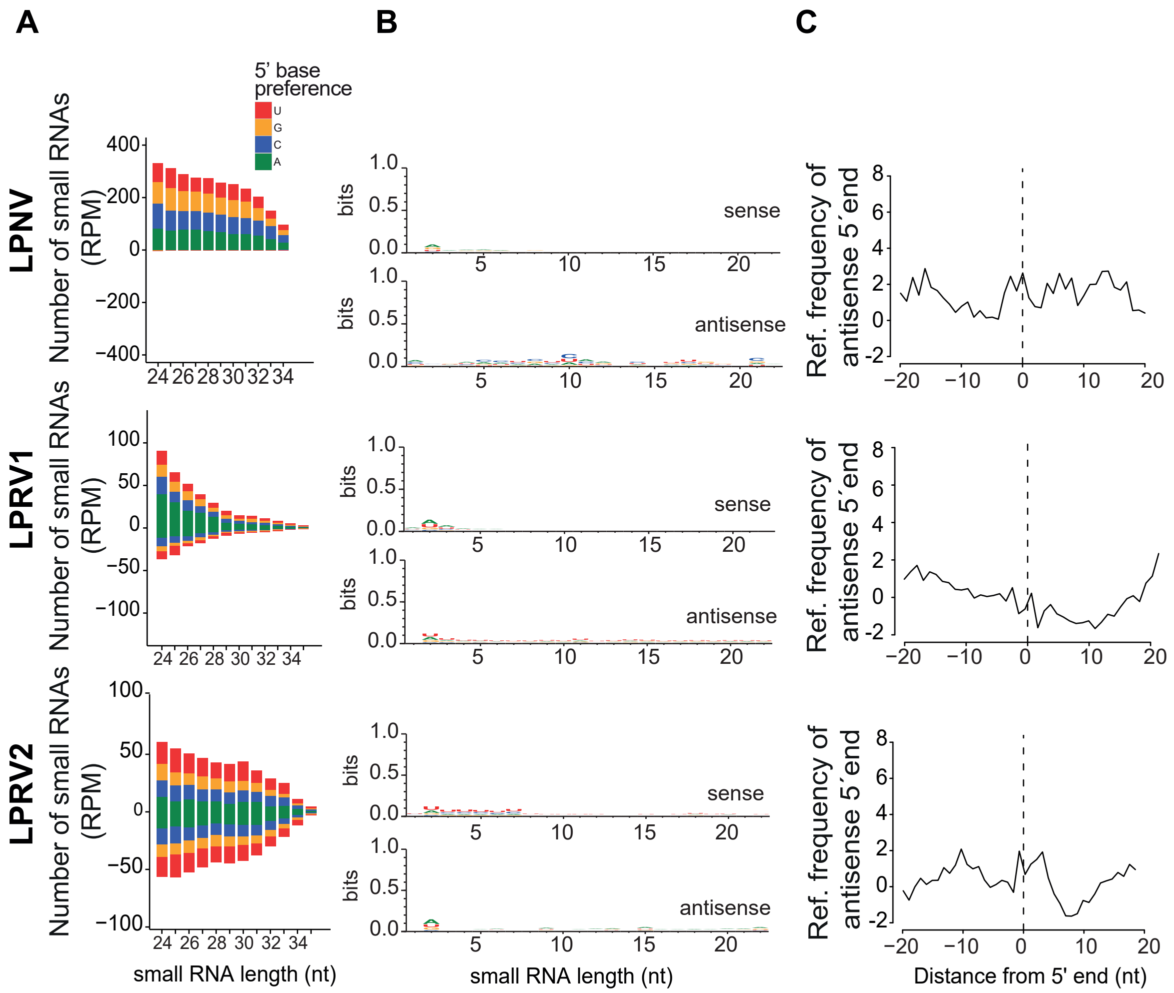

S3 Fig. Virus-derived small RNAs do not show canonical characteristics of piRNAs in *L. longipalpis*. Small RNAs derived from *Lutzomyia Piaui nodavirus* (LPNV), *Lutzomyia Piaui reovirus* 1 (LPRV1) and *Lutzomyia Piaui reovirus 2* (LPRV2) in *L. longipalpis* were analyzed for piRNA characteristics. **(A)** The size distribution of small RNAs in the 24-35 nt range considering each strand separately. 5' base preferences of small RNAs are indicated by color. **(B)** Nucleotide preferences for each position of virus-derived small RNAs between 24-30 nt are shown as a weblogo. **(C)** The relative frequency of overlap between 5' ends of small RNAs between 24-30 nt in opposite strands is shown.
