# Supplementary material for: The small non-coding RNA response to virus infection in the Leishmania vector Lutzomyia longipalpis
Source: PLoS Negl Trop Dis. 2018 Jun 4;12(6):e0006569. doi: 10.1371/journal.pntd.0006569 (PMC6002125; doi:10.1371/journal.pntd.0006569)
Supplement: S4 Fig — Size distribution of host small RNAs from L. longipalpis obtained from VSV-infected and control (Mock) sandflies at day 2, 4 and post blood feeding. 5’ base preferences of small RNAs are indicated by color. (PDF) [file pntd.0006569.s004.pdf]

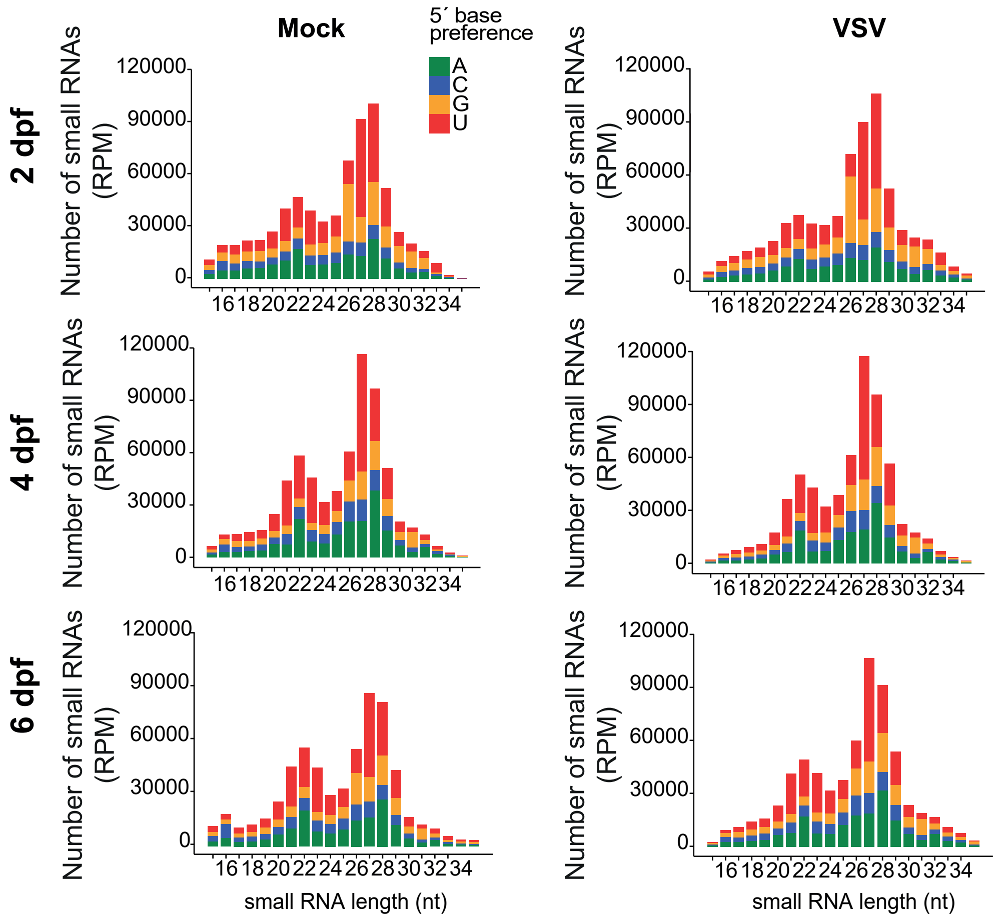

S4 Fig. The general profile of endogenous small RNAs does not change in response to VSV infection in *L. longipalpis.* Size distribution of host small RNAs from *L. longipalpis* obtained from VSV-infected and control (Mock) sandflies at day 2, 4 and post blood feeding. 5' base preferences of small RNAs are indicated by color.
