# Supplementary material for: The small non-coding RNA response to virus infection in the Leishmania vector Lutzomyia longipalpis
Source: PLoS Negl Trop Dis. 2018 Jun 4;12(6):e0006569. doi: 10.1371/journal.pntd.0006569 (PMC6002125; doi:10.1371/journal.pntd.0006569)
Supplement: S1 Table — (DOCX) [file pntd.0006569.s005.docx]

**S1 Table. Overview of small RNA libraries from *L. longipalpis***

| **SRA ID** | **organism** | **origin** | **sample** | **#total reads** | **#host reads** |
| --- | --- | --- | --- | --- | --- |
| SRR1803384 | *Lutzomyia longipalpis* | whole body | Mock  2 dpf | 10,679,412 | 9,852,586 |
| SRR1803386 | *Lutzomyia longipalpis* | whole body | Mock  4 dpf | 9,463,241 | 8,162,975 |
| SRR6429752 | *Lutzomyia longipalpis* | whole body | Mock  6 dpf | 8,766,757 | 6,787,370 |
| SRR5224249 | *Lutzomyia longipalpis* | whole body | VSV  2 pdf | 8,500,448 | 7,418,078 |
| SRR1803385 | *Lutzomyia longipalpis* | whole body | VSV  4 dpf | 8,109,613 | 7,285,842 |
| SRR5224248 | *Lutzomyia longipalpis* | whole body | VSV  6 dpf | 8,587,131 | 7,196,009 |
| SRR6429759 | *Lutzomyia longipalpis* | Lulo cells | - | 9,767,093 | 7,763,872 |
