# Supplementary material for: The small non-coding RNA response to virus infection in the Leishmania vector Lutzomyia longipalpis
Source: PLoS Negl Trop Dis. 2018 Jun 4;12(6):e0006569. doi: 10.1371/journal.pntd.0006569 (PMC6002125; doi:10.1371/journal.pntd.0006569)
Supplement: S2 Table — (DOCX) [file pntd.0006569.s006.docx]

**S2 Table. Oligonucleotides utilized in this study**

| **Target** | **Position** | **Sequence** |
| --- | --- | --- |
| VSV | Left | CCTTTAGAAGGGAATTGGAAGAA |
| VSV | Right | TCTGCCGACTTGATAGGATTG |
| *RpL32* | Left | GGCGTGTCTTGGAGTTGGA |
| *RpL32* | Right | TGTCCGTCGTCGCTTCAA |
| *Dicer-2* | Left | GCAATCCAATCGCATCACT |
| *Dicer-2* | Right | GCGGGAATGAATCTGGAAT |
| *AGO2* | Left | GGGCATAAGTGTGGGAAAGA |
| *AGO2* | Right | CAATGGAAATGTTGGCTTCA |
| *r2d2* | Left | GATCGAAGCAGGAAGCAAAG |
| *r2d2* | Right | GAACGTGAAGGATGGAGCAT |
| dsVSV | Left | AATACGACTCACTATAGGGTCGGATGCTTCCAGAACCAG |
| dsVSV | Right | TAATACGACTCACTATAGGGCAGAAGTGGAAGGCAGGGTT |
| dsAGO2 | Left | TAATACGACTCACTATAGGGAGATCAAACAGTTGTGCAGCGTG |
| dsAGO2 | Right | ATTTAGGTGACACTATAGAAGTGGGGCAACCAAGAAGAATTGA |
| dsFluc | Left | TAATACGACTCACTATAGGGAGAAACAATCCGGAAGCGACCAA |
| dsFluc | Right | ATTTAGGTGACACTATAGAAGTGTGACTGGCGACGTAATCCAC |
