# Supplementary material for: The small non-coding RNA response to virus infection in the Leishmania vector Lutzomyia longipalpis
Source: PLoS Negl Trop Dis. 2018 Jun 4;12(6):e0006569. doi: 10.1371/journal.pntd.0006569 (PMC6002125; doi:10.1371/journal.pntd.0006569)
Supplement: S3 Table — (DOCX) [file pntd.0006569.s007.docx]

**S3 Table. Accession numbers of siRNA pathway genes from *L. longipalpis***

| **Gene name** | **Gene ID (VectorBase)** |
| --- | --- |
| *Dicer-2* | LLOJ006509 |
| *AGO2* | LLOJ006148 |
| *r2d2* | LLOJ000693 |
